# Periodontal Procedure Intensity and Incident Tinnitus in Korean Adults: A Nationwide Cohort Study

**DOI:** 10.3390/medicina62081578

**Published:** 2026-08-17

**Authors:** Yu-Rin Kim, Minkook Son, Seon-Rye Kim, Byung-Jun Cho

**Affiliations:** 1 Department of Dental Hygiene, Silla University, Busan 46958, Republic of Korea; dbfls1712@silla.ac.kr; 2 Department of Physiology, College of Medicine, Dong-A University, Busan 49315, Republic of Korea; 3 Institute of Health Medical Education Convergence Research, Kangwon National University, Samcheok 25913, Republic of Korea; 4 Department of Paramedicine, Kangwon National University, Samcheok 25913, Republic of Korea

**Keywords:** periodontal disease, tinnitus, national health insurance, Korea, cohort study

## Abstract

*Background and Objectives*: This study investigated the association between periodontal procedure intensity, serving as an indirect proxy for periodontal disease burden, and incident tinnitus in Korean adults. *Materials and Methods*: Using the Korean National Health Insurance Service–National Health Screening Cohort (2002–2019), we included adults aged ≥40 years who had a diagnosis of periodontal disease (International Classification of Diseases, 10th Revision [ICD-10] code K05), had undergone relevant periodontal treatment, and had available health-screening data. Because direct clinical periodontal measures were unavailable, participants were classified according to treatment intensity into: a mild procedure group (scaling or root planing only) and a moderate-to-severe procedure group (subgingival curettage, extraction, periodontal flap surgery, bone grafting, or guided tissue regeneration). Incident tinnitus was defined as an ICD-10 H93.1 diagnosis recorded during two or more outpatient visits to a neurology or otolaryngology department to improve diagnostic specificity. Hazard ratios (HRs) and 95% confidence intervals (CIs) were estimated using Cox proportional hazards models adjusted for demographic, socioeconomic, cardiometabolic, and behavioral factors. *Results*: Among 132,808 participants (76,844 mild; 55,964 moderate-to-severe), the incidence rate of tinnitus was higher in the moderate-to-severe group than in the mild group (4.38 vs. 3.87 per 1000 person-years). After adjustment, the moderate-to-severe group showed a significantly higher risk of tinnitus (HR 1.24, 95% CI 1.17–1.30). These associations were consistent across sex- and age-stratified analyses and in sensitivity analyses using stricter tinnitus definitions. *Conclusions*: Greater periodontal procedure intensity was associated with a modestly higher incidence of tinnitus in this nationwide cohort. Because procedure intensity serves only as an indirect surrogate for periodontal disease burden, and because auditory confounders and short-term procedure-related factors were unavailable, causality and a specific inflammatory mechanism cannot be established. Further studies incorporating direct periodontal measurements, audiological data, and prespecified lag-time analyses are warranted to confirm these findings.

## 1. Introduction

Tinnitus is the perception of sound in the absence of an external acoustic source and is commonly described as ringing, buzzing, or roaring [1]. It is prevalent worldwide, with estimates ranging from approximately 6.6% to 25% among adults [1,2,3,4]. Tinnitus can substantially impair quality of life by disrupting sleep, concentration, and emotional well-being. Because effective pharmacological treatments remain limited [5], identifying potentially modifiable risk factors is of considerable clinical and public health importance.

The etiology of tinnitus is multifactorial and remains incompletely understood. In addition to hearing loss and noise exposure, epidemiological studies suggest that cardiovascular, metabolic, endocrine, and neuropsychiatric conditions may contribute to tinnitus risk [6,7,8,9]. Experimental and clinical studies also support a potential role for inflammation, particularly neuroinflammation, in the pathophysiology of tinnitus [10,11].

Periodontitis is a chronic inflammatory disease affecting the tooth-supporting tissues and has been associated with systemic inflammatory and vascular conditions [12]. Periodontal inflammation is characterized by elevated levels of inflammatory mediators, endothelial dysfunction, and impaired microcirculation [12,13,14]. Because the cochlea and auditory pathways may be vulnerable to vascular and inflammatory insults, periodontal disease has been hypothesized to contribute to auditory symptoms, including tinnitus; however, direct mechanistic evidence in humans remains limited [12,15,16,17].

Previous observational studies have reported associations between periodontal disease and hearing-related outcomes, including sudden sensorineural hearing loss and tinnitus [16,17,18]. However, most studies have focused on the presence of periodontal disease rather than on differences in treatment intensity. In large administrative health databases, direct clinical periodontal measures are generally unavailable. Procedure intensity can therefore serve as a pragmatic treatment-based indicator of periodontal disease burden [19,20,21]. However, it is not equivalent to clinical periodontal severity, because treatment escalation may also depend on clinician judgment, patient adherence, financial circumstances, access to dental care, and healthcare-utilization patterns.

Accordingly, we investigated whether periodontal procedure intensity was associated with incident tinnitus in a nationwide Korean cohort and examined whether the findings were consistent across subgroups defined by sex and age, as well as across alternative operational definitions of tinnitus.

## 2. Materials and Methods

### 2.1. Participant Selection

**Figure 1:**
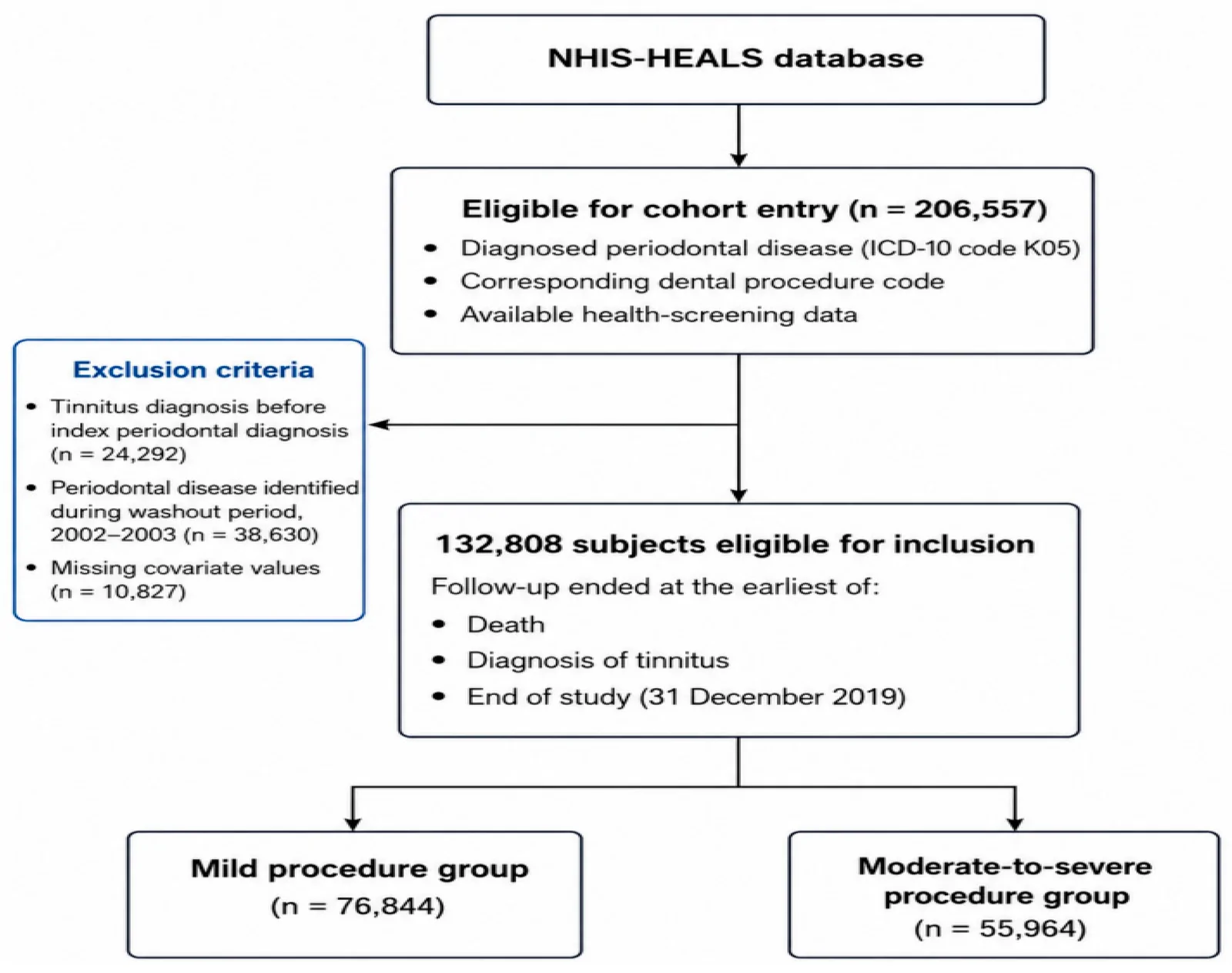
Flow diagram of participant selection.

The Korean National Health Insurance Service–National Health Screening Cohort (NHIS-HEALS) is a longitudinal cohort of individuals who participated in National Health Insurance Service health screenings. These biennial examinations are offered to adults aged 40 years or older. The study population was derived from NHIS-HEALS data collected between 1 January 2002, and 31 December 2019. Participants with an International Classification of Diseases, 10th Revision (ICD-10) code K05 and corresponding dental procedure codes were identified in the NHIS-HEALS database. Inclusion required at least one documented periodontal disease diagnosis (ICD-10 code K05), a corresponding periodontal procedure, and available health-screening data. A total of 206,557 individuals met these initial inclusion criteria. We then excluded participants with a tinnitus diagnosis recorded before the index periodontal diagnosis (n = 24,292), those whose periodontal disease was identified during the washout period from 1 January 2002, to 31 December 2003 (n = 38,630), and those with missing covariate values (n = 10,827). The final study population comprised 132,808 participants, of whom 76,844 were assigned to the mild procedure group and 55,964 to the moderate-to-severe procedure group. Figure 1 shows the participant selection process. Because claims data do not capture detailed clinical periodontal findings, the periodontal status of individuals without documented periodontal procedures could not be reliably determined; this population may range from periodontally healthy individuals to those with severe untreated periodontitis. We therefore did not use individuals without documented periodontal procedures as the reference group. Standardized audiometric data and detailed information on baseline hearing loss or deafness were not available in NHIS-HEALS; therefore, participants with pre-existing hearing impairment could not be excluded.

### 2.2. Definition of Periodontal Procedure Intensity

Periodontal disease was defined using ICD-10 code K05 together with specific periodontal treatment codes. Because clinical periodontal measures, including probing depth, clinical attachment loss, and radiographic bone loss were unavailable in the claims data [21], procedure categories were used as an indirect proxy for treatment intensity and underlying periodontal disease burden rather than as a direct measure of clinical severity. Under Korean National Health Insurance reimbursement rules, routine tooth scaling can be covered once per year only when accompanied by a periodontitis diagnosis code (K05). Thus, in clinical practice, a K05 code may be entered to support insurance coverage for scaling even when the primary purpose is preventive oral care.

On the basis of these reimbursement conditions, participants who received tooth scaling or root planing only, with no record of more intensive periodontal procedures, were categorized as the mild procedure group. Participants who underwent subgingival curettage, tooth extraction, periodontal flap surgery, bone grafting for alveolar bone defects, or guided tissue regeneration were categorized as the moderate-to-severe procedure group [20]. Tooth extraction was considered only among participants already included in the K05-defined periodontal cohort and only as one component of the procedure-intensity classification; it was not treated as a standalone indicator of periodontal severity, because extraction may be performed for nonperiodontal indications. More broadly, this claims-based classification reflects clinician-determined treatment escalation and may be influenced by biological disease burden, patient compliance, financial factors, access to dental care, and healthcare-utilization patterns. Exposure misclassification therefore cannot be excluded.

### 2.3. Outcome Definition: Tinnitus

Incident tinnitus was identified using ICD-10 code H93.1 and was defined as at least two outpatient visits to a neurology or otolaryngology department with a tinnitus diagnosis [18]. The repeated-visit requirement was chosen to improve diagnostic specificity in claims data and to reduce the influence of one-time provisional, incidental, or administrative coding; it should not be interpreted as a clinical requirement for more than one tinnitus evaluation. This specificity-oriented definition may reduce sensitivity by excluding patients who receive only one specialty outpatient assessment. Follow-up began on the date of the periodontal disease diagnosis and continued until tinnitus onset, death, or 31 December 2019, whichever occurred first. For sensitivity analyses, we applied increasingly stringent definitions restricted to outpatient visits to neurology or otolaryngology departments: Tinnitus 1 required ≥2 visits; Tinnitus 2 required ≥3 visits; and Tinnitus 3 required ≥4 visits with an H93.1 diagnosis. These definitions were intended to assess diagnostic specificity and the robustness of the findings rather than to classify tinnitus severity.

### 2.4. Covariates

Covariates were selected a priori on the basis of previous literature, biological plausibility, and availability in NHIS-HEALS. They were grouped into demographic characteristics, comorbidities, and health-screening variables. Demographic variables included age, sex, and income quartile; income quartile was derived from insurance premium-based household income and categorized into four levels. Comorbidities included ischemic heart disease, hypertension, dyslipidemia, diabetes, depression, cerebrovascular disease, and dementia. These conditions were identified using ICD-10 codes recorded within 1 year before the periodontal disease diagnosis. Hypertension was defined as ICD-10 codes I10 or I11 with at least one antihypertensive prescription per year, or a systolic blood pressure ≥ 140 mmHg or diastolic blood pressure ≥ 90 mmHg [22]. Diabetes was defined as ICD-10 codes E10-E14 with at least one antidiabetic prescription per year, or a fasting blood glucose level ≥ 126 mg/dL [23]. Dyslipidemia was defined as ICD-10 code E78 with at least one lipid-lowering prescription per year. Ischemic heart disease and cerebrovascular disease were identified using ICD-10 codes I20–I25 and I60–I69, respectively. Depression was defined using ICD-10 codes F32 and F33, and dementia using ICD-10 codes F00, F01, and F03. Health-screening variables included fasting blood glucose, systolic and diastolic blood pressure, body mass index, total cholesterol, smoking status (never, former, or current), current alcohol consumption (no or yes), and regular exercise frequency (none, 1–2 times/week, 3–4 times/week, or ≥5 times/week). Behavioral variables were obtained from the standardized health-screening questionnaire. Standardized audiometric measures, detailed baseline hearing-loss severity, occupational or recreational noise exposure, and detailed periprocedural exposure to analgesics or nonsteroidal anti-inflammatory drugs related to dental procedures were unavailable for covariate adjustment.

### 2.5. Statistical Analysis

All analyses were conducted using R version 4.3.0 (R Foundation for Statistical Computing, Vienna, Austria) [24] and SAS version 9.4 (SAS Institute Inc., Cary, NC, USA) [25]. Normality of continuous variables was assessed using the Shapiro–Wilk test and visual inspection of distributional plots. Baseline characteristics were compared between the mild and moderate-to-severe procedure groups using Student’s *t*-test for continuous variables and the chi-square test for categorical variables. Tinnitus incidence rates were calculated per 1000 person-years for each group during follow-up. Kaplan–Meier curves and the log-rank test were used to compare tinnitus-free survival. Cox proportional hazards models were used to estimate hazard ratios (HRs) and 95% confidence intervals (CIs) for incident tinnitus. Death was treated as a censoring event in the cause-specific Cox framework; competing-risk approaches, such as the Fine–Gray subdistribution hazard model, may provide complementary estimates in future analyses. The proportional hazards assumption was evaluated using Schoenfeld residuals and plots of the log cumulative hazard based on Kaplan–Meier estimates; no meaningful violations were identified. Multivariable models were adjusted for age, sex, income, ischemic heart disease, diabetes, hypertension, dyslipidemia, depression, cerebrovascular disease, dementia, body mass index, smoking status, alcohol consumption, and exercise. Subgroup analyses were performed by sex and age (<65 vs. ≥65 years), with the corresponding stratification variable excluded from each model. Statistical significance was defined as a two-sided *p* value < 0.05. Sensitivity analyses used alternative tinnitus definitions based on the number of outpatient visits. Formal interaction tests and lag-time exclusion analyses were not performed.

### 2.6. Ethical Considerations

Ethical approval was granted by the Institutional Review Board of Youngsan University (IRB No. YSUORB-202212-HR-122-02). The requirement for informed consent was waived because the study used de-identified secondary claims and health-screening data. The study was reported in accordance with the Strengthening the Reporting of Observational Studies in Epidemiology (STROBE) statement [26].

## 3. Results

### 3.1. Participant Characteristics by Periodontal Procedure Intensity

Participants in the mild procedure group (scaling/root planing only) had a mean age of 56.2 ± 7.7 years, compared with 57.3 ± 9.3 years in the moderate-to-severe procedure group. Although this between-group difference was statistically significant, its absolute magnitude was modest. Males comprised the majority of participants in both groups. In the mild procedure group, the proportion of participants increased across higher income quartiles, whereas in the moderate-to-severe procedure group, the largest proportion was in the third quartile. Diabetes, hypertension, cerebrovascular disease, and dementia were more prevalent in the moderate-to-severe procedure group, whereas dyslipidemia, ischemic heart disease, and depression were more common in the mild procedure group. Mean body mass index was 24.1 ± 2.9 kg/m^2^ in the mild procedure group and 24.0 ± 3.0 kg/m^2^ in the moderate-to-severe procedure group. Given the large cohort size, small absolute differences may yield statistically significant *p*-values; therefore, statistical significance should be interpreted alongside the magnitude and clinical relevance of the difference. Additional health-screening characteristics are presented in Table 1.

By the end of follow-up, tinnitus occurred more frequently in the moderate-to-severe procedure group across all operational definitions. Under the Tinnitus 1 definition, tinnitus occurred in 2740 participants (3.6%) in the mild procedure group and 2671 (4.8%) in the moderate-to-severe procedure group. Under the Tinnitus 2 definition, the corresponding numbers were 1885 (2.5%) and 1860 (3.3%), respectively. Under the Tinnitus 3 definition, tinnitus occurred in 1435 (1.9%) and 1422 (2.5%) participants, respectively. Across all three definitions, tinnitus was significantly more frequent in the moderate-to-severe procedure group than in the mild procedure group (all *p* < 0.05) (Table 1).

### 3.2. Risk of Incident Tinnitus According to Periodontal Procedure Intensity

**Figure 2:**
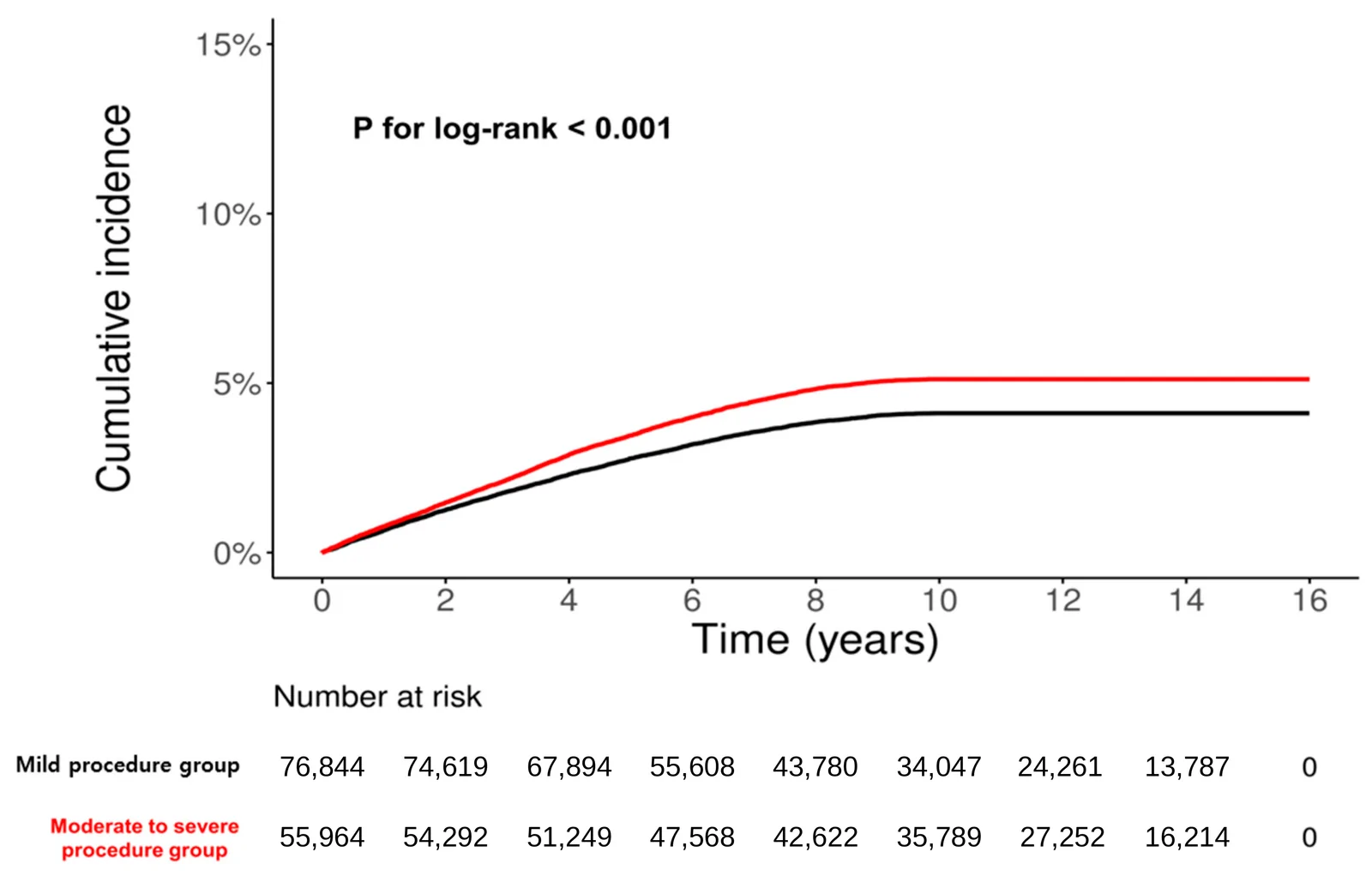
Kaplan–Meier curve for tinnitus-free survival according to periodontal procedure intensity. Black line: mild procedure group; red line: moderate-to-severe procedure group.

Tinnitus incidence rates were 3.87 and 4.38 per 1000 person-years in the mild and moderate-to-severe procedure groups, respectively. In Cox proportional hazards models, participants in the moderate-to-severe procedure group had a higher risk of incident tinnitus than those in the mild procedure group. The crude HR was 1.25 (95% CI: 1.19–1.32), and the adjusted HR was 1.24 (95% CI: 1.17–1.30) (Table 2). The Kaplan–Meier curve also showed lower tinnitus-free survival in the moderate-to-severe procedure group (log-rank *p* < 0.001) (Figure 2).

### 3.3. Subgroup Analyses

In sex-stratified analyses, tinnitus incidence remained higher in the moderate-to-severe procedure group among both males and females. Compared with the mild procedure group, the adjusted HR was 1.18 (95% CI 1.10–1.28) in males and 1.29 (95% CI 1.19–1.39) in females.

Age-stratified analyses similarly showed a higher tinnitus incidence in the moderate-to-severe procedure group in both age groups. Compared with the mild procedure group, the adjusted HR was 1.24 (95% CI 1.17–1.32) among participants aged <65 years and 1.19 (95% CI 1.07–1.33) among those aged ≥65 years (Table 2).

### 3.4. Sensitivity Analyses of Tinnitus Case Definitions

Under the second operational definition (Tinnitus 2), incidence rates were 2.64 and 3.01 per 1000 person-years in the mild and moderate-to-severe procedure groups, respectively. The moderate-to-severe procedure group had a higher risk of tinnitus, with a crude HR of 1.27 (95% CI 1.19–1.35) and an adjusted HR of 1.25 (95% CI 1.17–1.34). Under the third operational definition (Tinnitus 3), incidence rates were 1.99 and 2.29 per 1000 person-years, respectively. The crude HR was 1.28 (95% CI 1.19–1.37), and the adjusted HR was 1.26 (95% CI 1.17–1.36), again indicating a higher risk of tinnitus in the moderate-to-severe procedure group (Table 3).

## 4. Discussion

In this nationwide cohort of Korean adults with a documented periodontal diagnosis, greater periodontal procedure intensity was associated with a higher incidence of tinnitus. The association remained statistically significant after adjustment for measured demographic, socioeconomic, cardiometabolic, and behavioral covariates, and was consistent in sex- and age-stratified analyses and across alternative tinnitus definitions. These findings indicate an epidemiological association with treatment intensity and should not be interpreted as evidence that clinically measured periodontal severity directly causes tinnitus.

This study extends previous claims-based research by comparing levels of periodontal treatment intensity among individuals with a documented K05 diagnosis. Restricting the analysis to this diagnosed cohort may reduce some heterogeneity in dental care-seeking behavior compared with using individuals without documented dental care as a reference group presumed to be healthy. However, treatment intensity remains an indirect surrogate rather than a direct clinical periodontal measure, and the observed association may reflect both biological disease burden and patterns of clinical decision-making and healthcare utilization.

Systemic inflammation and neuroinflammation represent possible pathways linking periodontal disease and tinnitus, but the present data cannot directly test these mechanisms. Periodontitis is associated with elevated levels of inflammatory mediators, endothelial dysfunction, and impaired microcirculation [12,13,14], whereas experimental studies have implicated neuroinflammatory processes in tinnitus [11,27,28]. However, C-reactive protein (CRP), interleukin-6 (IL-6), and other inflammatory biomarkers were not available in our dataset. Moreover, a recent longitudinal human study reported no significant associations between plasma CRP, IL-6, or tumor necrosis factor receptor 2 and incident tinnitus [29]. Accordingly, the adjusted HR of 1.24 observed in the present study should not be attributed specifically to systemic inflammation; inflammation remains only one of several untested explanations.

The use of procedure intensity as a surrogate for periodontal severity warrants particular caution. Direct clinical measures, such as probing depth, clinical attachment loss, and radiographic bone loss, were unavailable. Treatment decisions may be influenced by patient adherence, financial considerations, access to dental care, clinician judgment, comorbidities, and willingness to undergo invasive procedures. In addition, tooth extraction is not specific to periodontitis. Although extraction was considered only within the K05-defined periodontal cohort and only as part of the overall procedure-intensity classification, the indication for each extraction could not be confirmed. Residual exposure misclassification is therefore possible, and both its direction and magnitude are uncertain.

Tinnitus was identified using repeated outpatient diagnostic codes recorded in neurology or otolaryngology departments. The primary requirement of at least two specialty visits was chosen to improve claims-based diagnostic specificity, not because two visits are clinically required to diagnose tinnitus. A single specialty visit may represent a valid diagnosis, particularly in healthcare systems in which patients are assessed and discharged after one outpatient appointment. Our approach may therefore fail to capture some valid cases and may limit direct comparability across healthcare systems. Requiring three or four visits in sensitivity analyses further increased specificity but likely reduced sensitivity. Visit frequency may also be influenced by symptom persistence and healthcare-seeking behavior; therefore, residual outcome misclassification remains possible.

The association was observed in both sexes and both age groups, with somewhat higher point estimates in females and participants younger than 65 years. These differences may reflect variation in baseline tinnitus risk, symptom perception, or healthcare-utilization patterns [30,31]. Because formal interaction tests were not performed, these subgroup differences should not be interpreted as evidence of effect modification.

The adjusted association was modest (HR 1.24). Given an effect size of this magnitude, residual confounding, exposure misclassification, and differences in healthcare utilization could plausibly explain part or potentially all of the observed association. The findings should therefore be interpreted as hypothesis-generating rather than conclusive evidence that periodontal treatment intensity or periodontal inflammation has a causal effect on tinnitus.

Several important limitations should be acknowledged. First, we did not include a definitively periodontally healthy control group. Under the Korean National Health Insurance system, routine scaling may be accompanied by a K05 diagnosis for reimbursement even when performed primarily for preventive care, whereas individuals without dental visits may include people with untreated periodontitis. We therefore restricted the analysis to individuals with a documented periodontal diagnosis and compared procedure-intensity categories. Nevertheless, reliance on administrative claims creates the potential for coding errors and exposure misclassification, including the nonspecific nature of tooth extraction and the influence of adherence, financial circumstances, access to dental care, and clinician treatment decisions. Second, important auditory and procedure-related factors were unavailable. Standardized baseline audiometry and detailed hearing-loss status could not be incorporated, and occupational or recreational noise exposure was not measured, although hearing loss and noise exposure are major risk factors for tinnitus [6,17]. The analytical dataset also did not permit detailed assessment of short-term postoperative analgesic exposure related to dental procedures. High-dose salicylates can precipitate tinnitus, and frequent use of non-aspirin NSAIDs and acetaminophen has been associated with a higher risk of incident persistent tinnitus [32]. In addition, dental handpieces generate substantial high-frequency noise [33], and temporomandibular disorders are associated with tinnitus [34]. Thus, periprocedural medication use, acoustic exposure, or temporomandibular strain may provide alternative explanations for at least some early auditory symptoms, although these mechanisms could not be directly evaluated in the present study. Third, we did not perform a lag-time sensitivity analysis excluding tinnitus events that occurred during the first year after the index date. Although participants with tinnitus before the index periodontal diagnosis were excluded, this restriction does not eliminate the possibility of reverse causation or acute procedure-related effects. Consequently, we cannot determine whether the association would persist after early events were excluded. Future studies should prespecify lag periods, including a 1-year lag, and consider competing-risk methods. Finally, inflammatory biomarkers such as CRP and IL-6 were unavailable, precluding mediation or mechanistic analyses; therefore, no causal inflammatory pathway can be inferred from this study.

Despite these limitations, this study has several strengths, including a large nationwide cohort, an extended follow-up period, adjustment for multiple measured clinical and behavioral factors, and sensitivity analyses using more stringent tinnitus coding definitions. Overall, the findings provide population-level evidence of an association between greater periodontal procedure intensity and incident tinnitus, although substantial uncertainty remains regarding residual confounding, exposure classification, early procedure-related effects, and biological mechanisms.

## 5. Conclusions

This nationwide cohort study found that greater periodontal procedure intensity was associated with a modestly higher incidence of tinnitus. Procedure intensity is an indirect claims-based surrogate and should not be equated with clinically measured periodontal severity. Because baseline hearing status, noise exposure, periprocedural medication use, and early procedure-related effects could not be fully addressed, and because a 1-year lag-time analysis was not available, causal inference is not warranted. The findings also do not establish systemic inflammation as the underlying mechanism. Further studies incorporating direct periodontal and audiological measurements, medication and noise-exposure data, inflammatory biomarkers, and prespecified lag-time analyses are warranted to confirm and elucidate the observed association.

## Figures and Tables

**Table 1:** Baseline characteristics of the study population.

Characteristics (n = 132,808)	Mild Procedure Group (n = 76,844)	Moderate-to-Severe Procedure Group (n = 55,964)	*p*-Value
Demographics			
Age (years)	56.2 ± 7.7	57.3 ± 9.3	<0.001
Sex (%)			<0.001
Male	42,373 (55.1)	34,817 (62.2)	
Female	34,471 (44.9)	21,147 (37.8)	
Income level (%)			<0.001
1st quartile	10,160 (13.2)	8656 (15.5)	
2nd quartile	15,564 (20.3)	12,587 (22.5)	
3rd quartile	21,736 (28.3)	16,746 (29.9)	
4th quartile	29,384 (38.2)	17,975 (32.1)	
Underlying disease			
Hypertension (%)	31,805 (41.4)	25,620 (45.8)	<0.001
Diabetes (%)	8724 (11.4)	7609 (13.6)	<0.001
Dyslipidemia (%)	22,580 (29.4)	13,466 (24.1)	<0.001
Ischemic heart disease (%)	8989 (11.7)	6031 (10.8)	<0.001
Cerebrovascular disease (%)	2476 (3.2)	2067 (3.7)	<0.001
Depression (%)	7372 (9.6)	4089 (7.3)	<0.001
Dementia (%)	474 (0.6)	435 (0.8)	<0.001
Health screening			
Body mass index (kg/m^2^)	24.1 ± 2.9	24.0 ± 3.0	<0.001
Systolic blood pressure (mmHg)	124.4 ± 15.3	127.2 ± 16.5	<0.001
Diastolic blood pressure (mmHg)	77.7 ± 10.3	79.1 ± 10.7	<0.001
Fasting blood glucose (mg/dL)	99.0 ± 24.5	100.6 ± 29.4	<0.001
Total cholesterol (mg/dL)	199.4 ± 37.2	198.0 ± 37.3	<0.001
Smoking (%)			<0.001
Never-smoker	52,072 (67.8)	34,571 (61.8)	
Former smoker	12,035 (15.7)	7056 (12.6)	
Current smoker	12,737 (16.6)	14,337 (25.6)	
Alcohol drinking (%)	35,598 (46.3)	26,400 (47.2)	0.002
Regular exercise (%)			<0.001
No	42,176 (54.9)	31,365 (56.0)	
1–2 times/week	19,996 (26.0)	13,884 (24.8)	
3–4 times/week	8972 (11.7)	5944 (10.6)	
≥5 times/week	5700 (7.4)	4771 (8.5)	
Outcomes			
Tinnitus 1 (%)	2740 (3.6)	2671 (4.8)	<0.001
Tinnitus 2 (%)	1885 (2.5)	1860 (3.3)	<0.001
Tinnitus 3 (%)	1435 (1.9)	1422 (2.5)	<0.001

Data are presented as mean ± standard deviation or n (%). Continuous variables are shown as rounded summary values, whereas *p* values were calculated using the values entered into the statistical analyses. Given the large sample size, small absolute differences may yield statistically significant *p* values and should not necessarily be interpreted as clinically meaningful.

**Table 2:** Hazard ratios and 95% confidence intervals for incident tinnitus according to periodontal procedure intensity.

All (n = 132,808)	Events	Follow-Up Duration (Person-Years)	Incidence Rate (Per 1000 Person-Years)	Hazard Ratio (95% Confidence Intervals)	
				Crude	Adjusted *
Mild procedure group (n = 76,844)	2740	707,255	3.87	1.00 (reference)	1.00 (reference)
Moderate-to-severe procedure group (n = 55,964)	2671	610,473	4.38	1.25 (1.19–1.32, *p* < 0.001)	1.24 (1.17–1.30, *p* < 0.001)
Male (n = 77,190)					
Mild procedure group (n = 42,373)	1340	394,265	3.40	1.00 (reference)	1.00 (reference)
Moderate-to-severe procedure group (n = 34,817)	1429	382,985	3.73	1.21 (1.12–1.30, *p* < 0.001)	1.18 (1.10–1.28, *p* < 0.001)
Female (n = 55,618)					
Mild procedure group (n = 34,471)	967	317,124	3.05	1.00 (reference)	1.00 (reference)
Moderate-to-severe procedure group (n = 21,147)	859	231,187	3.72	1.36 (1.26–1.46, *p* < 0.001)	1.29 (1.19–1.39, *p* < 0.001)
Age < 65 years (n = 109,473)					
Mild procedure group (n = 66,011)	1474	629,145	2.34	1.00 (reference)	1.00 (reference)
Moderate-to-severe procedure group (n = 43,462)	1274	503,901	2.53	1.20 (1.13–1.28, *p* < 0.001)	1.24 (1.17–1.32, *p* < 0.001)
Age ≥ 65 years (n = 23,335)					
Mild procedure group (n = 10,833)	569	84,693	6.72	1.00 (reference)	1.00 (reference)
Moderate-to-severe procedure group (n = 12,502)	803	112,277	7.15	1.15 (1.03–1.28, *p* = 0.01)	1.19 (1.07–1.33, *p* = 0.002)

* The model was adjusted for age, sex, income, hypertension, diabetes, dyslipidemia, ischemic heart disease, cerebrovascular disease, depression, dementia, body mass index, smoking, alcohol consumption, and exercise.

**Table 3:** Sensitivity analyses using alternative operational definitions of tinnitus.

All (n = 132,808)	Events	Follow-Up Duration (Person-Years)	Incidence Rate (Per 1000 Person-Years)	Hazard Ratio (95% Confidence Intervals)	
				Crude	Adjusted *
Tinnitus 2					
Mild procedure group (n = 76,844)	1885	715,352	2.64	1.00 (reference)	1.00 (reference)
Moderate-to-severe procedure group (n = 55,964)	1860	618,160	3.01	1.27 (1.19–1.35, *p* < 0.001)	1.25 (1.17–1.34, *p* < 0.001)
Tinnitus 3					
Mild procedure group (n = 76,844)	1435	719,657	1.99	1.00 (reference)	1.00 (reference)
Moderate-to-severe procedure group (n = 55,964)	1422	622,236	2.29	1.28 (1.19–1.37, *p* < 0.001)	1.26 (1.17–1.36, *p* < 0.001)

* The model was adjusted for age, sex, income level, hypertension, diabetes, dyslipidemia, ischemic heart disease, cerebrovascular disease, depression, dementia, body mass index, smoking, alcohol consumption, and regular exercise frequency.

## Data Availability

The data that support the findings of this study are available from the National Health Insurance Service Data Sharing Service (https://nhiss.nhis.or.kr (accessed on 10 October 2025)), but restrictions apply to their use. Access to the data requires approval from the National Health Insurance Service.

## Abbreviations

The following abbreviations are used in this manuscript:

NHIS-HEALS	Korean National Health Insurance Service–National Health Screening Cohort
ICD-10	International Classification of Diseases, 10th Revision
HR	hazard ratio
CI	confidence interval
